# Nano-Photonic Structures for Light Trapping in Ultra-Thin Crystalline Silicon Solar Cells

**DOI:** 10.3390/nano7010017

**Published:** 2017-01-13

**Authors:** Prathap Pathi, Akshit Peer, Rana Biswas

**Affiliations:** 1 Ames Laboratory, Microelectronics Research Center, Iowa State University, Ames, IA 50011, USA; prathap@nplindia.org; 2 Silicon Solar Cell Division, CSIR-National Physical Laboratory, Dr. K.S. Krishnan Road, New Delhi-110012, India; 3 Ames Laboratory, Microelectronics Research Center, Department of Electrical and Computer Engineering, Iowa State University, Ames, IA 50011, USA; apeer@iastate.edu; 4 Ames Laboratory, Microelectronics Research Center, Department of Physics and Astronomy, Department of Electrical and Computer Engineering, Iowa State University, Ames, IA 50011, USA

**Keywords:** nano-photonics, solar cell, light-trapping, scattering

## Abstract

Thick wafer-silicon is the dominant solar cell technology. It is of great interest to develop ultra-thin solar cells that can reduce materials usage, but still achieve acceptable performance and high solar absorption. Accordingly, we developed a highly absorbing ultra-thin crystalline Si based solar cell architecture using periodically patterned front and rear dielectric nanocone arrays which provide enhanced light trapping. The rear nanocones are embedded in a silver back reflector. In contrast to previous approaches, we utilize dielectric photonic crystals with a completely flat silicon absorber layer, providing expected high electronic quality and low carrier recombination. This architecture creates a dense mesh of wave-guided modes at near-infrared wavelengths in the absorber layer, generating enhanced absorption. For thin silicon (<2 μm) and 750 nm pitch arrays, scattering matrix simulations predict enhancements exceeding 90%. Absorption approaches the Lambertian limit at small thicknesses (<10 μm) and is slightly lower (by ~5%) at wafer-scale thicknesses. Parasitic losses are ~25% for ultra-thin (2 μm) silicon and just 1%–2% for thicker (>100 μm) cells. There is potential for 20 μm thick cells to provide 30 mA/cm^2^ photo-current and >20% efficiency. This architecture has great promise for ultra-thin silicon solar panels with reduced material utilization and enhanced light-trapping.

## 1. Introduction

Crystalline silicon solar cells are the dominant technology for solar panels, accounting for nearly 90% of the present market share. Crystalline silicon solar cells have achieved ~25% power conversion efficiency (PCE) in small area laboratory cells [1,2] and up to 22% in larger area panels. A very attractive feature of crystalline silicon (c-Si) technology is its high stability over several years, with a degradation rate typically less than 0.5% per year.

The silicon wafers utilized in solar panels are typically 180 μm thick. Hence, the material costs associated with thick silicon wafers are a considerable component of the system costs. It is of great interest to thin the c-Si wafers considerably, and employ recently developed light-trapping techniques [3] to absorb solar photons in thin layers. Moreover, thin silicon solar cells may be flexible and adapted to curved surfaces, increasing tremendously their range of application. Thus, the development of ultra-thin c-Si cells will be an important new technology direction that may have many significant technological impacts.

**Figure 1:**
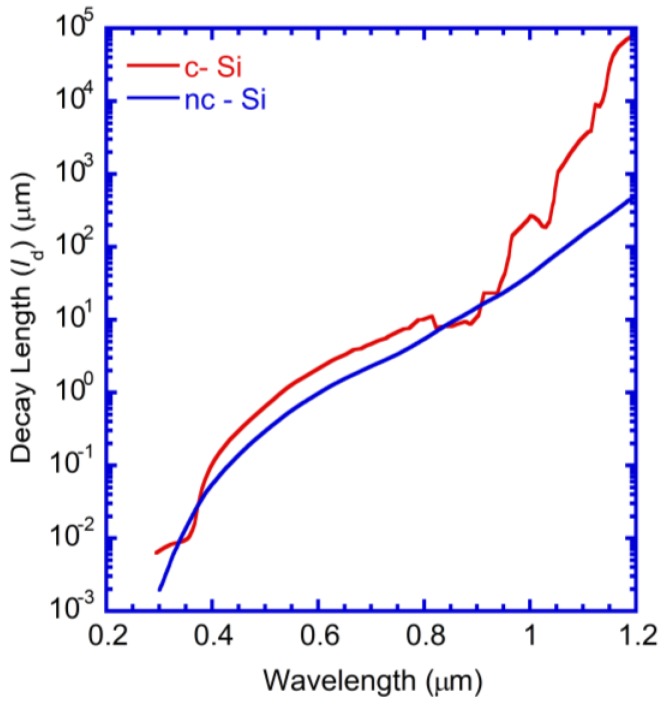
Photon absorption length as a function of wavelength for crystalline silicon (c-Si) and nano- crystalline silicon (nc-Si) (using the complex refractive index (*n*, *k*) parameters of Reference [26]).

A major technical hurdle is that the absorption length (*l_d_*(λ) = 4π*Im*(*n*(λ))/λ) of photons (Figure 1) rapidly increases at wavelengths near the band edge (1100 nm or 1.12 eV), where *n*(λ) is the complex refractive index of Si [4]. At the near-infrared wavelength, λ = 900 nm and *l_d_*(λ) is 10 μm for Si and grows exponentially for longer wavelengths, exceeding the thickness of the absorbing layer in thin cells. Such photons cannot be effectively absorbed in thin Si layers. It is necessary to employ light trapping techniques to increase the path length of long wavelength red and near-infra-red (IR) photons. A very attractive solar cell architecture for thin amorphous silicon (a-Si:H) and nano-crystalline Si (nc-Si) solar cells [5,6,7,8,9,10,11,12] is to utilize a periodically corrugated back-reflector and grow a conformal solar cell on top of this structure such that all layers have the periodic corrugation. This architecture traps light through (i) strong diffraction leading to a dense mesh of wave-guided modes propagating in the plane of the structure and (ii) propagating surface plasmon modes at the semiconductor-metal interface where the light intensity is considerably enhanced. These effects have resulted in measured enhancement of short circuit current (*J_SC_*) exceeding 30% in periodically corrugated nc-Si cells.

A similar diffractive structure has been proposed for thin c-Si solar cells [13,14,15] consisting of a periodic array of silicon nanocones both on the front and back of the structure combined with a perfect electrical conductor serving as a back reflector. Such thin c-Si cells, with a thickness of just ~2 μm, are predicted to have absorption and photo-current near the Lambertian limit [16,17]. However, preliminary experimental solar cells with this architecture [14] have considerably lower PCE (8%), much lower than predicted [13]. The reason for this is that the photonic crystal is composed of corrugated Si surfaces, where the corrugation leads to a large surface area and surface recombination of photo-excited carriers. The increased recombination losses outweigh the advantages of optical absorption enhancements. A triangular array of dielectric nanospheres on a flat Ag layer has been alternatively proposed [18,19] to be a high performing back reflector, which preserves the flatness of the c-Si layers and is also amenable to fabrication.

Thus, an effective solar light-trapping architecture is to utilize diffractive photonic crystal surfaces that involve flat silicon layers, that minimize carrier recombination losses, and use periodic arrays of insulating materials for diffractive effects. In this paper, we develop and design a practical light-trapping architecture utilizing photonic crystals of insulating materials that preserves electronic quality of the interfaces. There are analogies with the proposed architecture of Ingenito et al. [20] where a front surface was composed of textured ”black-silicon” combined with the back surface having a random pyramidal silicon texture and a distributed Bragg reflector (DBR), with predicted absorption near the Lambertian limit. We design an alternative architecture based on periodic nanostructured arrays (rather than random features) and use a simpler metallic back reflector rather than the DBR. The present architecture avoids the alkaline texturization process, which is performed for absorption enhancement of c-Si solar cells. Enhancement of long wavelength absorption in thin Si through rear surface plasmons has been demonstrated for planar Si absorbing layers with Ag nanoparticles on a detached back surface reflector [21,22], an architecture that preserves high electronic quality of interfaces.

Light trapping has also been applied to crystallized silicon on glass (CSG) cells by texturing of the glass substrate [23] leading to *J_SC_* of 29 mA/cm^2^–29.5 mA/cm^2^ for up to 3.5 μm thick poly-Si absorber layers. This is slightly lower than the Lambertian limit (34 mA/cm^2^). Front texturing of the glass increases light path through randomization but may not approach the full randomization of light within the absorber layer as suggested by the Lambertian limit. We have theoretically [24] and experimentally [25] studied periodic texturing of the glass in solar cells and found gains that were significantly lower than those possible from texturing the absorber layer itself. As described by Varlamov et al., light trapping in CSG cells has also been implemented via etch-back texturing of the poly-Si itself and does extremely well at long wavelengths (>600 nm) but not in the broad band spectrum, resulting in *J_SC_* of 29.5 mA/cm^2^ for a 3.6 μm cell.

## 2. Results

### 2.1. Approach and Structure

We design a practical light trapping architecture where all silicon interfaces are planar and demonstrate a high degree of light trapping, close to the Lambertian limit, that is achieved by photonic crystals of non-absorbing insulating materials. This architecture is concurrently expected to have superior electronic properties, comparable to conventional high-efficiency silicon solar cells. Our solar architecture differs from the previous work of Wang et al. [13] where the silicon was part of the photonic crystal leading to much higher surface recombination of photo-excited carriers.

**Figure 2:**
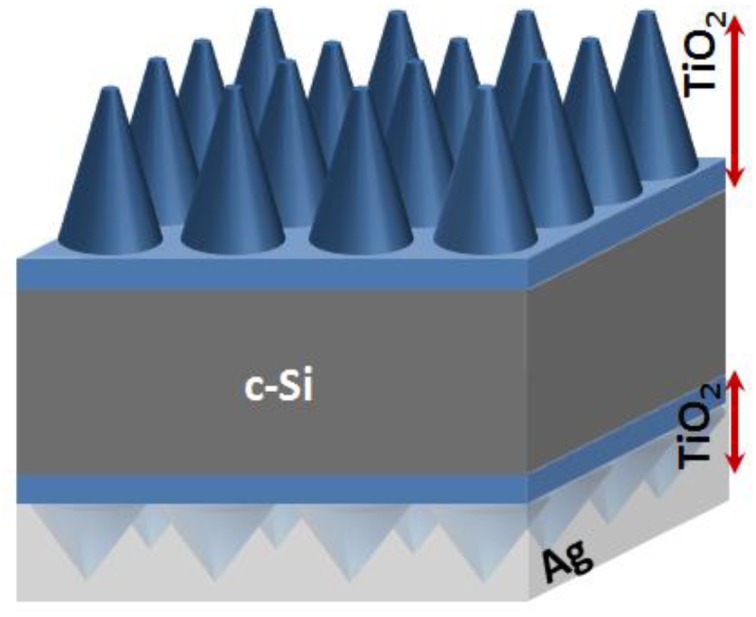
Proposed solar architecture consists of thin flat spacer titanium dioxide (TiO_2_) layers on the front and rear surfaces of silicon, nanocone gratings on both sides with optimized pitch and height, and rear cones are surrounded by Ag metal reflector.

Our proposed solar cell architecture (Figure 2) consists of (1) an upper photonic crystal array of dielectric titania nano-cones arranged in a triangular lattice, with height *d*_0_ and pitch a; on (2) a passivating layer of titania (thickness *d*_1_); (3) the flat silicon absorber layer (thickness *d*_2_); (4) another passivating layer of titania (thickness *d*_3_); followed by (5) a lower photonic crystal array of titania nanocones with height *d*_4_ and pitch a; that is coated with (6) a metallic (Ag) reflecting layer. The upper photonic crystal has two functions. It diffracts incoming light into the thin absorber layer and realizes a gradual transition from air to the dielectric, thereby reducing impedance mismatch and reflection loss of incoming light back to air. The lower photonic crystal is effective in diffraction of long wavelength light that reaches the back of the cell, but has a much smaller effect on shorter wavelengths that are absorbed within the upper portion of the absorber layer. Without the metallic back-reflector, the long wavelength photons can transmit and escape through the back of the cell. For computational convenience, the front and lower photonic crystal arrays have the same pitch. As required in the high efficiency silicon solar cells, both interfaces of silicon are passivated with thin titanium dioxide (TiO_2_) layers. The passivating layers are flat minimizing interfacial recombination. These unique structures have great promise for the fabrication of high efficiency thin c-Si solar cells using PERC (passivated emitter rear contact), PERL (passivated emitter with rear locally diffused), or PERT (passivated emitter, rear totally diffused) configurations.

It is necessary to systematically design the solar cell structure for optimal performance. We first design optimum photonic crystal based silicon solar architectures with rigorous vectorial simulations using the scattering matrix method, where Maxwell’s equations are solved in a plane wave basis, i.e., in Fourier space, for both polarizations of the incident wave. Maxwell’s equations in real space are converted to equations for each frequency ω, in Fourier space:

∇×E=iωH

(1) ∇×H=iωε(r)E

In Fourier space, the solutions of Maxwell’s equations are independent for each incoming frequency (ω) or incoming wavelength λ.

Experimentally measured wavelength-dependent complex dielectric functions ε(λ) for Si, Ag, and TiO_2_ are utilized. The solar cell is divided into layers in the z direction. Within each layer of the structure (Figure 2), the dielectric function ε(*x*, *y*) is a function of the spatial coordinates (*x*, *y*) but not of *z*. This allows the dielectric function in each layer to be expanded in a two-dimensional basis of reciprocal lattice vectors *G*, providing the Fourier components of the dielectric function ε(*G*). Similarly, the electric fields and magnetic fields (*E*(*G*), *H*(*G*)) are expanded in Bloch waves. Within each layer, Maxwell’s equations are solved in Fourier space in an eigenvalue expansion [27,28] to obtain the electric and magnetic fields in each layer. A transfer matrix is used to relate the *E* and *H* fields within each layer. Maxwell’s equations are integrated with the continuity boundary conditions throughout the unit cell to obtain the scattering matrices of each layer and the entire structure. From the scattering matrix we find the total reflectance R (including diffracted beams) and transmission T (which is 0) at each incident wavelength. The absorption at each wavelength is then *A* = 1 − *R* − *T*. Details have been covered in previous publications [29]. We characterize solar cell architectures by their weighted absorption <*A_w_*>,

(2) $$<A_{w}>=\int_{\lambda_{1}}^{\lambda_{2}}A(\lambda )\frac{dI}{d\lambda }d\lambda ,$$

and short circuit current *J_SC_*,

(3) $$J_{SC}=\frac{e}{hc}\int_{\lambda_{1}}^{\lambda_{2}}\lambda A(\lambda )\frac{dI}{d\lambda }d\lambda$$

Here, d*I*/dλ is the incident solar spectrum. We assume ideal internal quantum efficiency, i.e., absorption of each incoming photon generates an electron-hole pair. We have been very successful in designing optimized thin silicon periodic nano-photonic structures with this rigorous approach [5,29]. 

### 2.2. Design of Light Trapping Architecture Methods and Structure

It is of much interest to harvest photons in thin Si-layers/foils over a broad-band of wavelengths below the Si band edge (1.12 μm). The large index mismatch between silicon and air causes significant reflection losses for flat structures. This can be reduced using ergonomic tapered nanostructures which grade the refractive index from Si to the air value. Furthermore, the nanostructures diffract in-coming light and increase the photon path length within the absorber layer.

**Figure 3:**
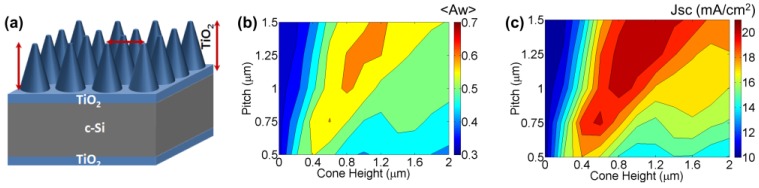
(**a**) Cell structure used for optimization of texture parameter using simulations. The structure consists of thin flat TiO_2_ layers on the front and rear surfaces of flat silicon (1 μm). The cones are only on the front surface without rear cones and Ag metal reflector; (**b**) Weighted absorption, <*A_w_*> and (**c**) Short-circuit current density, *J_SC_*, as a function of cone height for different pitch values. Figure shows optimum pitch >750 nm and cone height >500 nm with an increasing trend of tolerance of optimum cone height at larger pitch.

We performed a systematic set of optimizations for all parameters in this architecture (Figure 3). To conceptually understand the enhancement mechanism, it has been convenient to perform optimization by disassembling some of the components and starting with the simpler structure of two TiO_2_ layers passivating silicon. The optimal passivating layer thicknesses was found to be 60 nm and 50 nm on the front and rear Si-surfaces, respectively, close to the expected quarter-wavelength for red light (λ ≈ 600 nm).

After this step the front nano-cones were added and their structure was optimized. Finally, the back cones were added followed by the metallic back reflector. All structural parameters were optimized to achieve maximum anti-reflection and light trapping over the broad-band solar spectrum (300 nm–1100 nm) of interest for a silicon cell. We chose TiO_2_ for the dielectric layers due to its dual characteristic of passivating p-type and n-type silicon surfaces and ability to transport electrons.

#### 2.2.1. Front Nanocone Array

Light trapping in the Si absorber layer is achieved through the generation of wave-guided modes propagating parallel to the interface [30,31]. In order to achieve the maximum light absorption, ensuring anti-reflection and light trapping, the structural requirements (pitch, aspect ratio) of the front and rear cone morphology were studied individually, and then combined. The front cone height was first optimized to achieve maximum absorption in silicon. The structure simulated consists of a front texture comprised of flat silicon (1 μm thickness), front spacer layer, front cones, and rear spacer layer without cones (Figure 3a). The optimum cone height was studied for different array pitch values ranging from 0.25 μm to 1.5 μm in steps of 0.25 μm. As shown in Figure 3b,c, the weighted absorption <*A_w_*> and photo-current *J_SC_* have a strong inter-dependence of the pitch and cone height. The absorption and *J_SC_* are low at small pitch values (below 0.5 μm) irrespective of the cone height. There is a region of high absorption where <*A_w_*> ≈ 0.61 (in the orange region Figure 3a), where the cone height ranges from 0.6 to 1.2 μm, for pitch values of 0.75 to 1.5 μm, with the optimized cone height increasing with the pitch. *J_SC_* shows similar trends with a correspondingly wider region of high *J_SC_* ≈ 20.7 mA/cm^2^ (Figure 3c), where the optimized cone height increases from 0.6 to 1.4 μm as the pitch increases from 0.75 to 1.5 μm. As expected from mode coupling studies [31], the optimum pitch values are of the same order as the range of wavelengths that need to be absorbed. The simulation reveals it is advantageous to use a pitch larger than 0.75 μm, since optimum height has a tolerance of >±100 nm and does not demand high precision processing equipment, an advantage for manufacturing. For example, a pitch of 1.25 μm is coupled with a cone height of 700 nm–900 nm.

In the next optimization sequence, we kept the front cone geometry fixed (with pitch 750 nm and height 600 nm), introduced back cones, and varied the height of the back cones. For computational convenience, we chose the same pitch for the front and back photonic crystals. The height of the back cone was optimized and found to be ~200 nm.

Even with the back cones there is significant transmission through the structure. To completely eliminate this transmission, the back cones were embedded in the silver back reflector; this eliminated transmission and provided a rear mirror to enhance absorption and photo-current.

#### 2.2.2. Absorption Enhancement

**Figure 4:**
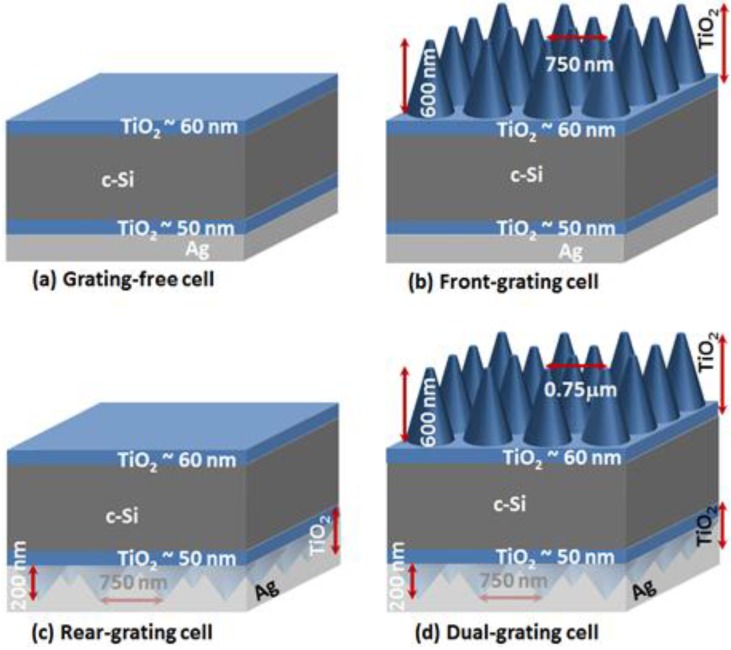
Sequence of light trapping structures on flat silicon. (**a**) Grating-free cell with thin layers of 60 and 50 nm on front and rear surface, respectively, and flat Ag back-reflector; (**b**) Front-grating cell with only front cones of height of 600 nm and a pitch of 750 nm and flat Ag back-reflector; (**c**) *Rear-grating cell* with only rear cones of height of 200 nm and a pitch of 750 nm and corrugated Ag back-reflector; (**d**) *Dual-grating cell* with a combination of front and rear cones with optimized parameters used in ‘b’ and ‘c’ with corrugated Ag back-reflector.

A sequence of light-trapping photonic crystal structures (Figure 4), that were systematically built-up on a flat silicon surface, were analyzed to understand the enhancement contributions from the different components. The four sequential configurations (Figure 4) are the (a) Grating-free cell; (b) Front-grating cell; (c) Rear-grating cell, and (d) Dual-grating cell with a metallic back-reflector.

**Figure 5:**
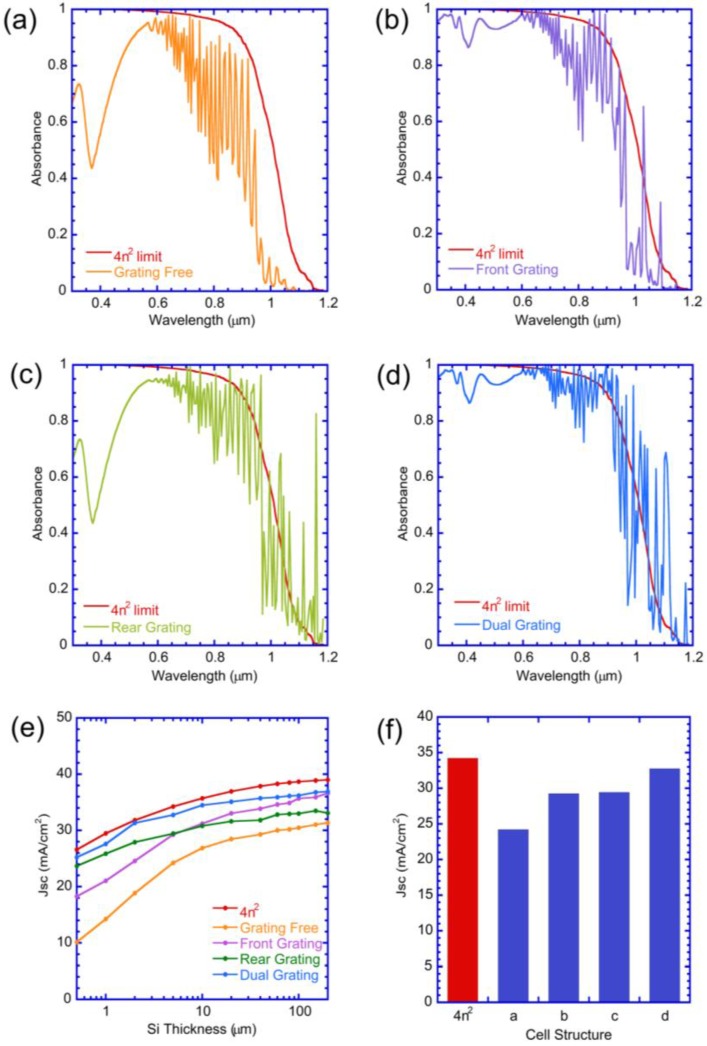
Comparison of absorption spectra of planar silicon with 4*n*^2^ absorption limit for different light trapping configurations shown in Figure 2 such as (**a**) *grating-free* *cell*, (**b**) *front-grating cell*, (**c**) *rear-grating cell*, and (**d**) *dual-grating cell* using the optimized grating parameter for 2 μm silicon; (**e**) Comparison of *J_SC_* of planar cell for different light trapping configurations shown in Figure 2 with respect to silicon thickness using optimized grating parameters for 2 μm silicon; (**f**) *J_SC_* of the cell for a particular thickness of 2 μm for four different configurations.

Scattering matrix simulations were performed for each solar architecture (Figure 4) with a flat c-Si absorber layer of 2 μm. The optimized value for the photonic crystal arrays were obtained, and the wavelength-dependent absorbance of each architecture (Figure 5a–d) was compared with the Lambertian (4*n*^2^) limit, in which the light is completely randomized [32] within the absorber layer.

The starting flat solar cell (Figure 4a) with a flat back reflector (Figure 5a) displays poor absorbance at short wavelengths (λ < 550 nm), wave-guiding modes in the red and near-IR (between 600 nm and 900 nm), absorbance considerably below the Lambertian limit, and a photo-current of 15 mA/cm^2^ (Figure 5e,f). The addition of the front photonic crystal (Figure 4b) reduces the reflectance loss, coupling light more effectively into the cell, and increases the absorbance at shorter wavelengths (λ < 550 nm) to near the Lambertian limit (Figure 5b). The overall absorbance at longer wavelengths (λ > 800 nm) is still low, indicating inadequate light trapping.

Alternatively, when the back photonic crystal is added to the flat solar cell (Figure 4c), the near-IR absorbance (λ > 800 nm) is considerably improved, approaching the Lambertian limit due to the dense mesh of waveguided modes (Figure 5c). However, the absorbance at short wavelengths (λ < 550 nm) is still low, similar to the flat cell, since these blue-green photons are reflected away from the solar cell. This emphasizes that the front grating is particularly beneficial in reducing reflectance loss and coupling short λ photons into the solar cell. The rear photonic crystal affects only longer-wavelength near-IR photons that reach the back reflector without being absorbed in the absorber layer and generates enhanced absorption at the Lambertian limit (λ > 800 nm).

The optimized front- and rear-photonic crystals were combined (Figure 4d) to generate a high absorbance over the entire wavelength range (Figure 5d). The absorbance and photo-current (Figure 5e,f) are very close to the Lambertian limit. As observed in previous studies [5,13] the absorbance exceeds the Lambertian limit at the specific wavelengths where wave-guiding in the plane of the structure occurs [33,34].

The dense mesh of wave-guiding modes generates absorption maxima from the diffraction resonances which are observed when the phase difference between modes reflected from the front and rear surfaces are multiples of 2π or *k_z_* = *m*π/*d*, *k_z_* is the z-component of the wave-vector [5]. For a triangular lattice, any reciprocal lattice vector *G* has components *i*(2π/*a*) and (2*j* − *i*)(2π/*a*)/√3, respectively, where *i* and *j* are integers. Incident light with wave-vector *k*_||_ is diffracted according to *k*_||_′ = *k*_||_ + *G*, since *k_z_* = *m*π/*d*, *k_z_*^2^ + *k*_||_^2^ = *n*(λ)^2^(ω/*c*)^2^ and wave-guiding occurs at resonant wavelengths given by:

(4) $$\lambda (i,j,m)=\frac{2\pi n(\lambda )}{{\left[\left(i^{2}+\frac{1}{3}{\left(2j-i\right)}^{2}\right){\left(\frac{2\pi }{a}\right)}^{2}+{\left(\frac{m\pi }{d}\right)}^{2}\right]}^{1/2}}$$

where *i*, *j* and *m* are integers. A dense mesh of wave-guided modes occurs in the long λ region (Figure 5), for our choice of parameters, where the wavelength inside silicon λ/*n*(λ) is smaller than the pitch (a) of the cones, resulting in large values of *i*, *j* and *m*. The phase coherence of waves is assumed at each interface. Accordingly, the resonant wavelength at which waveguide mode occurs is given by Equation (3).

The diffraction resonances result in propagation of wave-guided modes in the plane of the absorber layer that increases the path length and results in significant enhancement in the absorption at the wavelengths (Figure 5b–d) and enhanced *J_SC_*.

It is of interest to study the enhancement and photo-current *J_SC_* as a function of silicon thickness (Figure 5e) for the different solar cell architectures. The front cones are more effective at higher c-Si thicknesses (>40 μm), providing a substantial increase from the grating-free flat solar cell. The rear cones are more beneficial at lower thickness (<10 μm) in increasing the computed *J_SC_* (Figure 5e) from the flat case, but their benefit levels off at larger thicknesses. At the smaller thicknesses, diffraction induced by the rear cones improved the photon absorption at longer wavelengths. As discussed by Bermel et al. [35], the modes diffracted by the rear cones improves the coupling of electromagnetic field with diffraction modes as c-Si thickness is reduced.

The highest performing architecture is the dual-grating cell (Figure 4d) for all Si-thicknesses. The relative enhancement in *J_SC_* of the dual-photonic crystal cell with respect to the flat cell is 94% for the cell thickness of 1 μm as compared to 11% for a 200 μm thick cell. Figure 5f, shows the progression of *J_SC_* for a silicon thickness of 2 μm for different dielectric photonic crystal arrays. The flat solar cell has *J_SC_* = 18.9 mA/cm^2^. The front-grating improved the absorption and *J_SC_* to 24.6 mA/cm^2^, while the rear-grating alone improved it further to *J_SC_* of 27.9 mA/cm^2^. The dual-grating cell resulted in the *J_SC_* of 31.3 mA/cm^2^, which is very close to the 4*n*^2^ limit of 31.8 mA/cm^2^. The enhancement in <*A_w_*> and *J_SC_* are found to be 69% and 66%, respectively, in comparison to the grating-free cell. The *J_SC_* difference between Lambertian and the dual PC enhanced cell increases with thickness while the enhancement factors decrease with thickness. The dual nanocone arrays offer great promise to trap light in ultra-thin c-Si (<2 μm) and collect suitable currents.

We varied the aspect ratio of the front and rear nano-cones by varying their base radii, *R*, for a different pitch, *a*. We found that *R*/*a* ≈ 0.4 provides the best absorption, consistent with previous results for photonic crystal enhanced absorption [24,36]. With this geometry, there is a small flat section of the titania surface in between neighboring nano-cones.

#### 2.2.3. Variation with Angle of Incidence

**Figure 6:**
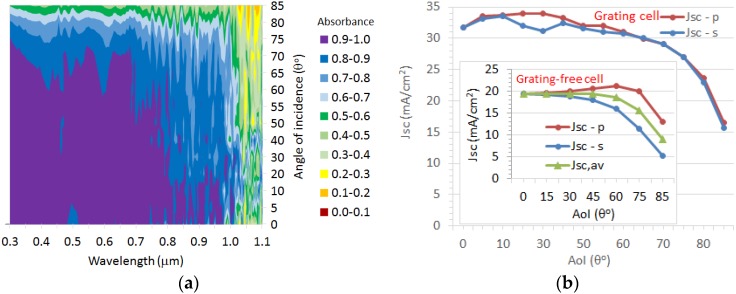
(**a**) Absorption spectra of the dual-grating cell (c-Si thickness = 2 μm) and (**b**) corresponding *J_SC_* of the cell as a function of angle of incidence (AoI) in the range 0°–85° in steps of 5° (inset shows the *J_SC_* of grating-free cell for p- and s-polarization and its average). The average absorption is more than 80% over a wide wavelength band. *J_SC_* is independent of polarization and is less influenced until the AoI reaches 70°, showing the omni-directionality of the nanocone grating structures.

Strong light absorption over a wide range of incident angles is crucial for the optimum performance of a solar cell during the entire daytime operating hours. Omni-directional absorbance is highly beneficial in capturing diffuse sunlight. The cell response was simulated for the optimized dual photonic crystal (PC) light-trapping architecture as a function of angle of incidence (AoI) (θ) for both incident polarizations. Figure 6a shows the contour plot of angle-dependent absorption of the 2 μm thick c-Si substrate with optimized surface nanostructures for different incident angles (in the azimuthal *x*–*z* plane) over the entire wavelength range of the solar spectrum. The absorbance exceeds 0.9 at an angle θ of 10° and is nearly constant above 0.8 until θ ≈ 70°, above which the absorption decreases. Figure 6b shows the predicted photo-current for both p-and s-polarizations over a wide range of θ. *J_SC_* is similar for both polarizations at large θ. Interestingly, the photo-current *J_SC_* has a maximum at θ ≈ 30° for p-polarization, while it has a maximum for 10° for s-polarization, so that these photonic crystal arrays perform better when the angle of incidence is somewhat away from the normal direction, and the incident light is nearly parallel to the surface of the nano-cones. This is advantageous for solar collection with fixed (non-tracking) solar panels, where the sun sweeps across the sky. The average *J_SC_* for both polarizations has a maximum of about 33.7 mA/cm^2^ at θ ≈ 10° and is over 30 mA/cm^2^ for θ > 70°. A similar trend was observed for the 1D or 2D silicon grating structures [37] and nano-photonic conformal nc-Si solar architectures [5]. For the grating-free cell, *J_SC_* for the p-polarization peaks at an θ ≈ 60° as a result of the Brewster angle, which reduces reflectance to zero at the front surface (inset of Figure 6b). Averaging both polarizations results in a constant *J_SC_* until θ ≈ 60°, after which the absorption and photo-current suffer sharply. The substantial increase of *J_SC_* for the nanocones over wide range of θ is due to the improved in-coupling of light by the PC array as discussed by Heine and Morf [37] for silicon gratings with respect to the incident angle of light.

The wide-angle light trapping and polarization independent characteristics of the nanocone arrays are due to their smooth graded-index profile and their gradual variation of optical density coupling which is better with incident light. The grating structures outperform the biomimetic silicon nanostructures [38] or silicon gratings with low-aspect ratio [39].

#### 2.2.4. Field Distribution

**Figure 7:**
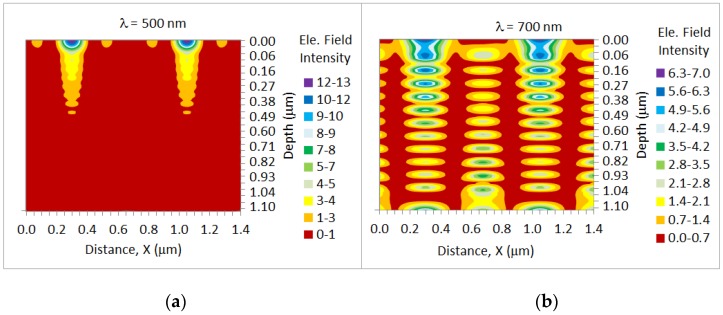
Electric field intensity distribution across the silicon absorber layer at an incident wavelength of (**a**) 500 nm and (**b**) 700 nm for the dual-grating cell with optimized parameters for the front and rear nano-cone arrays. The incident electric field intensity is normalized to 1.

The photonic crystal structures are effective in exciting wave-guided modes at wavelengths in the order of, or slightly larger than, the pitch of the arrays, according to the wave-guiding condition of the round-trip phase difference from the top and bottom being a multiple of 2π (Equation (3)). The transverse electric (TE) (*x*-polarized) modes in the c-Si of a thickness of 2 μm are simulated in a cross section of the solar architecture. The field distribution of the mode (Figure 7) shows the electric field intensity |*E*|^2^ at an incident wavelength of 500 nm (Figure 7a) and 700 nm (Figure 7b) (typical for the long λ regime) for the dual-photonic crystal cell with the optimized light trapping architecture. At λ = 500 nm, the incident photons are effectively absorbed at the top of the cell (Figure 6a) and do not interact with the rear nanostructure. Since the absorption length (*l_d_*) of light at 750 nm exceeds the thickness of the cell, the incident photons reach the rear photonic crystal array and both front and rear arrays are effective in diffraction. Standing waves are produced in the silicon layer by the nanocone arrays (Figure 7b). The separation between the maxima of the standing waves in the *z*-direction is the expected value λ/2*n*, which is close to 100 nm. This separation between maxima increases as the incident wavelength increases. In the case of nanocone array architecture (Figure 7b), there are regions of high field concentration at the top of the layer, where the field intensity is enhanced by a factor of ~7, which is one order more than that of its grating-free counterpart.

The guided mode in the dual photonic crystal structure propagates in the *x*-direction planar to the silicon layer, with a wavelength controlled by the pitch (*a* ≈ 750 nm) of the structure. For the flat cell, the guided modes cannot be excited by plane wave illumination from air, since the light line lies above the light line for the dielectric.

#### 2.2.5. Parasitic Losses

One of the critical questions is the magnitude of the parasitic losses within the metal electrode at the back of the cell, especially at long wavelengths, and the predicted enhancements when all parasitic losses are accounted for. Since the Ag electrode has *n*_2_ = *Im*(*n*) > 0, there are losses in the silver at longer wavelengths. Qualitatively, these losses are expected to be more severe for thinner cells where more light reaches the back-reflector. In the previous sections, the calculated wavelength dependent absorption, *A*(λ) included contributions from the Ag [5,24]. To de-convolute the contribution of the absorption from only the Si layer, it was necessary to extract the electric fields *E*(*r*) and magnetic fields *H*(*r*) in real space by transformation of the computed fields in Fourier space [24]. The absorption rate *R* per unit volume at the position *r* is:

(5) $$R=\frac{1}{8\pi }\omega Im\left(\varepsilon (\omega )\right){\left|E(r)\right|}^{2}$$

The absorption *A* is obtained by integrating over the volume *V* of the absorber layer:

(6) $$A=\frac{1}{V}\int \frac{4\pi n_{1}n_{2}}{\lambda }{\left|E(x,y,z)\right|}^{2}dxdydz$$

Such a procedure is computationally laborious and not well suited for the present scattering matrix method where Maxwell’s equations are solved in Fourier space rather than real space. In fact, such procedures are more amenable to real space solvers such as the finite difference time domain (FDTD) method. The use of Fourier space has great advantages for parallelization and computational efficiency at the expense of not directly calculating the real space fields and separating the losses. Nonetheless, we have implemented this procedure of extracting the real space fields and we separately calculate the losses only in the silicon. More than 1000 CPU hours are needed in a multi-processor environment utilizing 64 processors.

**Figure 8:**
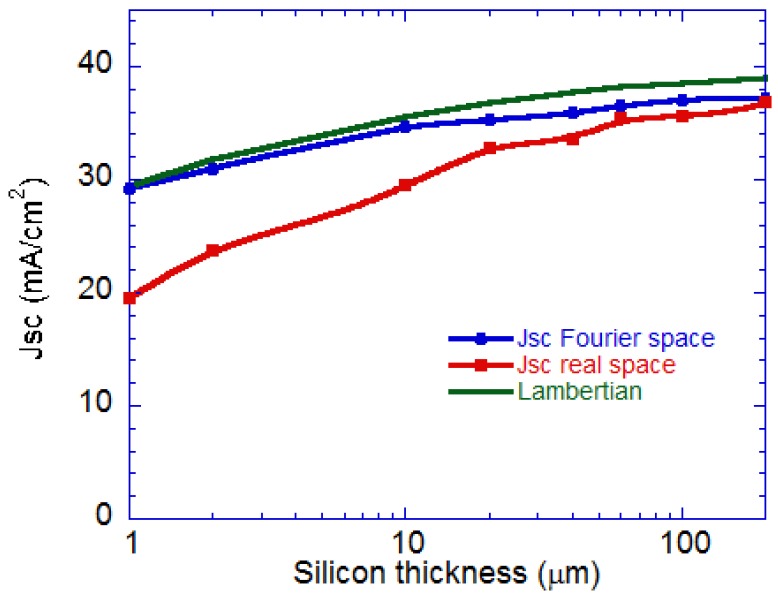
Comparison of the simulated short-circuit photo-current *J_SC_* as a function of the thickness of the Si absorber layer in the real-space method utilizing only the absorption in the Si layer, compared to the Fourier space method which includes the absorption in all layers. The difference between the real-space and Fourier space results is the parasitic absorption. The Lambertian limit is shown for comparison.

We simulated the absorption and *J_SC_* (Figure 8) with this real-space method as a function of the Si-absorber layer thickness and compared it with the previous Fourier space simulation that includes the absorption in the metal. The absorption and *J_SC_* is lower with the real-space method due to parasitic absorption in Ag. The difference between the real space and Fourier space is largest for the thinner absorber layers, but nearly negligible for thicker Si absorber layers (Figure 8). The difference between the photo-current by the real-space and Fourier space methods is the parasitic absorption, which varies from 1% for 200 μm Si to 15% for 10 μm and 33% for the thinnest 1 μm Si absorber. *J_SC_* reaches ~24 mA/cm^2^ for the 2 μm Si after accounting for parasitic losses. In comparison, <*A_w_*> is 0.5% to 25% lower as the Si thickness is decreased from 200 μm to 1 μm, respectively. This trend is to be expected since the red and near-IR wavelengths are absorbed in thicker cells and do not reach the back reflector, which consequently has negligible effect on parasitic absorption. A large fraction of the incident light reaches the back reflector in thin cells, increasing the parasitic absorption. Parasitic losses are due to ohmic losses in the structured Ag-dielectric interface. In principle, the parasitic absorption may be reduced by the use of a conductive distributed back reflector (DBR) [20,29] with flat interfaces, but the wavelength bandwidth of high reflectance may be smaller.

## 3. Discussion

Flat interfaces of silicon with passivating titania are expected to have a minimum density of surface defects with a high degree of passivation and electronic quality in comparison to that of the textured or patterned silicon surfaces. The photonic crystal arrays reside within the titania that control light trapping and can be easily fabricated by spraying titania gels on silicon and nano-imprinting the gel in a roll-to-roll method, viable for large scale production. Micro-transfer molding techniques using polydimethylsiloxane (PDMS) templates are suitable for small area laboratory cells. Additionally, the titania layers could be cured during the metallization step, where the metal top contacts are fabricated to the silicon layer. This reduces the additional substrate heating during the deposition step of the antireflection/passivation layer. The spacer layer thickness can be controlled by the processing parameters of pressure, temperature, etc., during imprinting. Our study demonstrates a large tolerance of the optimum design to grating parameters, which does not impose stringent conditions over the processing parameters. The grating structure allows for the use of thinner silicon substrates and avoids the physical or chemical etching or patterning processes that are used for light management in the standard silicon solar cell fabrication. The imprint process is proved to be attractive on plastic substrates for mass production [40].

After accounting for parasitic losses in the thin cells (Figure 8) we obtain predicted short circuit currents *J_SC_* of ~30 mA/cm^2^ (24 mA/cm^2^) for 20 μm (2 μm) thick cells. Utilizing the highest reported cell parameters of *V_oc_* = 0.74 V and fill factor (FF) of 0.827 for the Panasonic c-Si cell [2,41], we predict the efficiency to be ~20.2% (14.7%) for a 20 μm (2 μm) thick cell, which reduces Si material utilization by ~15 (compared to a 300 μm cell). This suggests that a ~20 cell μm may yield acceptable efficiency at significantly lower cost.

This work is significantly different from our previous dual photonic crystal solar architecture [24] which used (i) an organic absorber layer; (ii) a polymer lens array on glass for focusing; and (iii) a nanostructured organic layer, none of which are present here.

An alternative to the periodically structured photonic crystals is the use of a random texture on both the front and back surface [20] that randomizes light distribution in the material, a feature that has been extensively utilized in thin amorphous silicon cells.

An issue of much practical importance is that solar panels are subjected to harsh environmental conditions. To protect such panels with nanocone arrays we anticipate using a front glass protective sheet that is commonly employed in most solar panels. Thus, the nanocones are not in direct contact with the outside environment and are expected to have high stability. We have performed simulations with a protective glass sheet that shows the enhancements are similar to those in this paper.

This work mainly targets the state-of-the-art crystalline silicon cell structures (i.e., PERC/PERL/PERT) in the industrial environment irrespective of silicon thickness. These advanced structures require efficient passivation on both surfaces. Rear surface/back surface field (BSF) is contacted through via-holes, which are generally made using screen printer/laser ablation followed by metal filling. Therefore, the charge collection is ensured through via-holes filled with metal, and not through the TiO_2_ layer.

### Proof of Concept Demonstration of TiO_2_ Nanocone Array Fabrication

**Figure 9:**
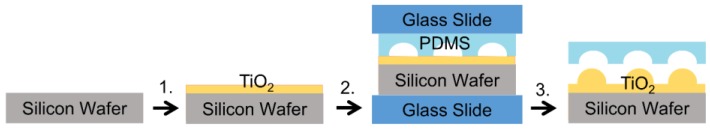
Schematic of the TiO_2_ nanoimprinting process. (**1**) TiO_2_ film is spin-coated on silicon substrate using a precursor titanium diisopropoxide bis(acetylacetonate); (**2**) The polydimethylsiloxane (PDMS) stamp having nanocups is placed on the spin-coated film with patterned side facing the film. The whole assembly is sandwiched between two glass slides and held together with binder clips (not shown here); (**3**) After keeping at ~170 °C for 15 min, the binder clips are released to reveal the inverse of PDMS nanocups on the TiO_2_ film.

We have developed a nano-imprinting procedure to demonstrate the feasibility of fabricating nano-cone arrays of titania on silicon substrates, and we illustrate a pathway for experimental fabrication of the simulated light-trapping structures. Briefly, the process started with a polycarbonate master pattern having nanocone gratings in triangular lattice with a period of ~750 nm and height ~150 nm. The master pattern was used to replicate the inverse of the nanocone array on a polydimethylsiloxane (PDMS) mold using soft lithography, using a procedure described in our previous publication [42]. The resulting PDMS mold with the inverse nanocup array was then used to imprint the TiO_2_ film. We adopted the process in Chen et al. [43] with slight modifications to fabricate the TiO_2_ film. We spin-coated 0.45 M titanium diisopropoxide bis(acetylacetonate) on a clean silicon wafer at 3000 rpm for 30 s. The film was then imprinted under elevated temperature and pressure with the PDMS mold using the process illustrated schematically in Figure 9 with more details in the following Section 4.

**Figure 10:**
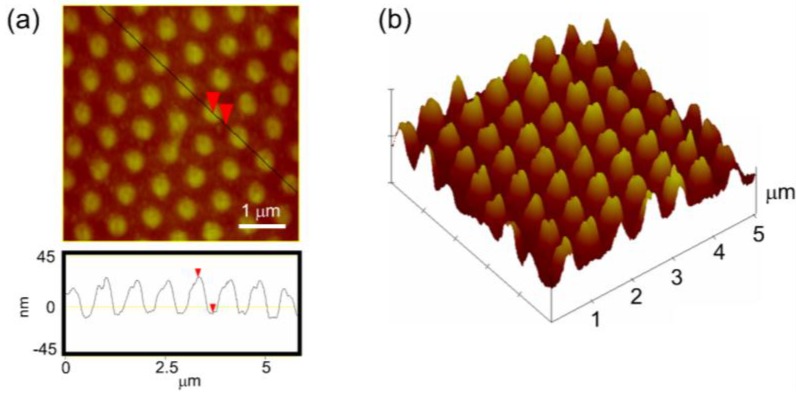
(**a**) Atomic force microscopy (AFM) image of the periodic array of nanocones imprinted on TiO_2_ film. The AFM line scan shows the periodicity at ~750 nm and the average height of nanocones at ~35 nm; (**b**) Three-dimensional view of the structure showing titania nanocone arrays.

Figure 10a shows the atomic force microscopy image of the nanoimprinted TiO_2_ film fabricated using soft lithography. The atomic force microscopy (AFM) line scan and three-dimensional view (Figure 10b) shows the well-replicated regular array of nanocones on TiO_2_ with a period of ~750 nm and average height of ~35 nm. It is to be noted that the feature height can be varied easily using the initial PDMS stamp of different nanocup depth. Since the TiO_2_ material is inherently hard and compact, the shallower stamp has difficulty in penetrating deeper into the film. However, the PDMS nanocups of much higher depths of the order of ~1 micron will yield nanocone gratings with height >500 nm–600 nm which is of interest to solar cell fabrication for efficient light trapping based on simulation results. The similar imprinting procedure has been successfully implemented by our group in nanoimprinting various polymer films such as polystyrene [40] and poly(l-lactic acid) [44].

## 4. Materials and Methods

The solar cell is divided into layers in the z direction. Within each layer of the structure (Figure 2), the dielectric function ε(*x*, *y*) is a function of the spatial coordinates (*x*, *y*) but not of *z*. This allows the dielectric function in each layer to be expanded in a two-dimensional basis of reciprocal lattice vectors *G*, providing the Fourier components of the dielectric function ε(*G*). Similarly, the electric fields and magnetic fields (*E*(*G*), *H*(*G*)) are expanded in Bloch waves. Within each layer, Maxwell’s equations are solved in Fourier space in an eigenvalue expansion. A transfer matrix is used to relate the *E* and *H* fields within each layer. Maxwell’s equations are integrated with the continuity boundary conditions throughout the unit cell to obtain the scattering matrices of each layer and the entire structure. From the scattering matrix we find the total reflectance *R* (including diffracted beams) and transmission *T* (which is 0) at each incident wavelength. The absorption at each wavelength is then *A* = 1 − *R* − *T*. This computational algorithm is executed on massively parallel computing clusters (using 192 to 256 processors) with each wavelength/frequency sent to a different processor, which considerably speeds the design time. The advantage of this scattering matrix approach is that a real-space grid is not needed, avoiding complications of large memory requirements for substrates.

Experimentally, the nanocones are fabricated using nanoimprint lithography (NIL) of sol-gel derived TiO_2_ layers. The PDMS mold was generated from the polycarbonate master. The precursor solution 0.45 M titanium diisopropoxide bis(acetylacetonate) was spin-coated on a clean silicon wafer at 3000 rpm for 30 s. The PDMS mold was placed such that the patterned side faced the spin-coated titania film and the whole assembly was sandwiched together in two glass slides using binder clips, which served to apply adequate pressure for nanoimprinting. After keeping the assembly at 170 °C for 15 min, the binder clips were released and the PDMS stamp was subsequently lifted to reveal the nanoimprinted film. The sample was then sintered in a furnace at 550 °C for 15 min. The nanoimprinted substrate was then immersed in 50 mM TiCl_4_ solution for 30 min at 70 °C and washed with deionized (DI) water, followed by annealing at 550 °C for 30 min. The purpose of annealing is to harden the nanoimprinted TiO_2_ film.

The imprinting process is more industrial friendly for roll-to-roll (R2R) or large area fabrication. The evolution of R2R or roll-to-plate (R2P) makes the NIL process cheaper and compatible with the existing fabrication lines in the photovoltaic (PV) industry [45].

## 5. Conclusions

We have designed a highly absorbing thin film crystalline Si based solar architecture, using periodically patterned front and rear photonic crystals. In contrast to previous approaches, we utilize dielectric photonic crystals, ensuring that the c-Si absorber layer has completely flat interfaces, which is expected to result in high electronic quality and low carrier recombination. The mechanism for enhanced absorption is the generation of a dense mesh of wave-guided modes at near IR wavelengths, propagating in the Si absorbing layer. An optimized structure consists of a triangular lattice of front nanocones of titania with a height of 600 nm and pitch of 750 nm, although other ranges of height/pitch combinations can provide similar results, such as a 1000 nm pitch with a nanocone height of 800 ± 100 nm. The nanocone height and pitch are strongly inter-related. The back reflector consists of another periodic array of nanocones of titania with similar pitch as the front array, embedded with a silver back reflector. For thin c-Si absorber layers (<2 μm), we achieve an absorption and photo-current enhancement exceeding 100%. For thick c-Si absorber layers, the enhancement is up to 20%. The absorption has approached the Lambertian limit at small thicknesses (<10 μm) and is only slightly lower by (~5%) at larger thickness of 200 μm. Although the absorption at any particular wavelength can exceed the Lambertian limit, the absorption averaged over all wavelengths and the photo-current is below the Lambertian value. The limiting Lambertian value, when averaged over all wavelengths, is an aspect for fundamental studies.

Parasitic losses are significant (>25%) for ultra-thin Si layers below 2 μm thickness, but decrease rapidly as the thickness of the Si absorber layer increases, and are just ~1%–2% for thick (>100 μm) cells. Electric field distributions demonstrate waveguiding modes and high intensity focusing regions within the absorber layer. The strong enhancements predicted by simulation should be tested by fabrication of such nano-photonic structured solar cells. The proposed structure is very promising for high performance flexible, ultra-thin Si solar cells, providing a pathway for low cost high performing silicon solar modules which should be valuable to researchers in this area.
